# Bilateral Pneumothorax and Subcutaneous Emphysema following Endoscopic Retrograde Cholangiopancreatography: A Rare Complication

**DOI:** 10.1155/2010/894045

**Published:** 2010-08-11

**Authors:** Fotios Sampaziotis, Alan Wiles, Syed Shaukat, Richard J. Dickinson

**Affiliations:** Hinchingbrooke Hospital, Hinchingbrooke Park, Huntingdon, Cambs PE29 6NT, UK

## Abstract

Endoscopic Retrograde Cholangiopancreatography (ERCP) is a widely used diagnostic and therapeutic modality in the management of biliary and pancreatic disease. Some of the complications of the procedure, although rare, may carry significant morbidity and mortality risks. We describe the case of a 68-year-old female who underwent elective ERCP for ductal stone clearance. Immediately postprocedure, the patient developed subcutaneous emphysema and bilateral pneumothoraces. Further imaging revealed the presence of free intra-abdominal air. The patient made a very quick recovery after bilateral chest drain insertion and no further intervention was required. We propose that pneumothorax, pneumomediastinum, and subcutaneous emphysema during ERCP, in the absence of duodenal perforation may be explained by leakage of air from a site of low resistance such as the sphincterotomy site, or as a result of copious Valsalva manoeuvres performed by a patient tolerating the procedure poorly.

## 1. Introduction

ERCP is a widely used diagnostic and therapeutic modality in the management of biliary and pancreatic disease. Some of the complications of the procedure, although rare, may carry significant morbidity and mortality risks [1, 2]. We describe a rare case of bilateral pneumothoraces complicating ERCP in the presence of extraluminal abdominal air.

## 2. Case Presentation

**Figure 1:**
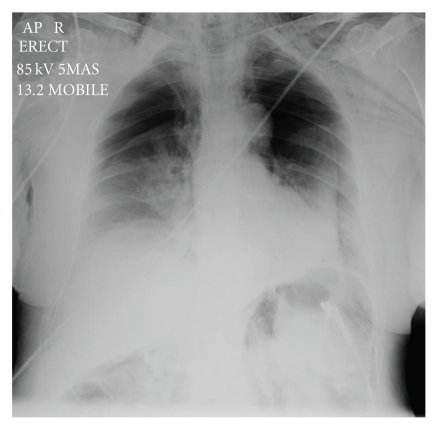
Chest X-Ray prior to chest drain insertion. Note the bilateral pneumothoraces.

**Figure 2:**
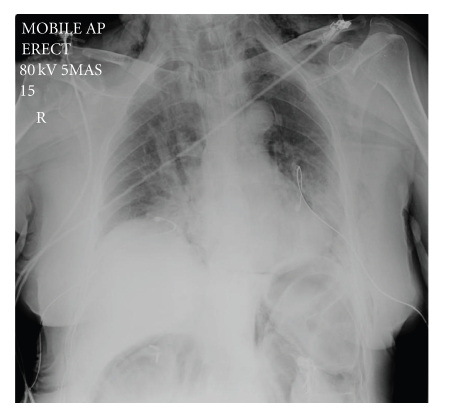
X-Ray post chest drain insertion. Note the presence of extraluminal air in the abdomen.

**Figure 3:**
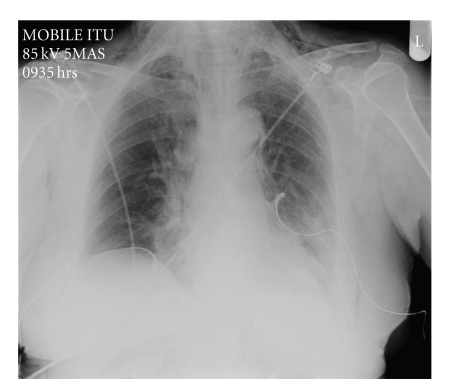
Chest X-Ray prior to chest drain removal.

A 68-year-old female patient was admitted as an elective patient for ductal stone clearance under midazolam sedation. Previous extraction had failed due to the size of the calculi and at that time sphincterotomy with biliary stent insertion was performed. At repeat ERCP, the sphincterotomy was extended and two calculi were removed. Immediately postprocedure, the patient became profoundly tachypnoeic and hypoxic. Physical examination revealed extensive subcutaneous emphysema and poor air entry bilaterally. The patient was transferred to HDU, where chest X-ray revealed extensive bilateral pneumothoraces and pneumomediastinum (Figure 1). Abdominal imaging (X-ray) revealed evidence of extra luminal air, pneumoperitoneum, and pneumoretroperitoneum. The patient was stabilized with bilateral chest drain insertion and made a rapid recovery (Figure 2). Antibiotic treatment was not required other than one single dose which was administered empirically immediately post procedure. By the third day of admission, the pneumothoraces had completely resolved (Figure 3) and chest drains were removed. The patient was discharged the following day.

## 3. Case Discussion

Only three cases of bilateral pneumothoraces complicating ERCP have been reported in the literature [3–5]. Unilateral pneumothorax, although rare, has been described in several cases. In all previous reports, the presence of air in the pleural cavity has been accompanied by pneumoperitoneum, pneumoretroperitoneum, and pneumomediastinum. The suggested mechanism is leakage of luminal air, tracking from the retroperitoneal space to the peritoneum, pleural space, mediastinum, and subcutaneous tissue. The spread of air takes place possibly through deep fascial planes [6], but a case of porous diaphragm syndrome has also been described recently [5]. The most common cause for leakage of luminal air during ERCP is duodenal perforation [7]; however, any site of low resistance like an ulcer or tumour may serve as a “release” valve during insufflation. [5, 6]. Three distinct types of ERCP-related perforations are described: (a) guidewire-related perforations, (b) periampullary perforations during sphincterotomy, and (c) perforations that are remote from the papilla [8, 9].

The above pathophysiological mechanisms may explain the constellation of symptoms and findings described in this case as a result of intraluminal air escaping in the abdomen. The process underlying the release of intraluminal air may be explained by the clinical picture. The patient made a remarkably quick recovery post chest drain insertion and remained completely asymptomatic until discharge. Only one dose of antibiotics was administered. The pneumothoraces, pneumomediastinum, pneumoretroperitoneum, and pneumoperitoneum had fully resolved by the third day of admission without the need for any further intervention.

This rapid clinical improvement would not be expected in the case of a duodenal perforation by the duodenoscope. The majority of cases of perforation post ERCP, remote from the papilla (oesophageal, gastric, duodenal), require surgery. All reported cases were symptomatic and required antibiotic cover [9–11]. Guided by the clinical picture and limited by the patient's wishes, who refused cross-sectional tomography oesophageal, gastric or duodenal perforation was excluded on clinical grounds. Although no further imaging was obtained due to the limitations of the specific case, we believe that the investigation of choice to exclude perforation would be a cross-sectional tomography study of the abdomen, which should always be part of the diagnostic workup in similar cases when possible.

The rapid clinical improvement of the patient could be better explained by a small leak of air arising from a site of low resistance, which in this case is most likely to be the sphincterotomy site, or a guidewire-related perforation. However, we note that most reported cases of sphincterotomy or guidewire related perforations are symptomatic and require broad-spectrum antibiotics, biliary and duodenal decompression [8, 9, 12, 13]. 

There is a second possible explanation regarding the mechanism leading to the patient's presentation and findings. This is based on our clinical observations during the procedure. In a sphincterotomy leak, the onset of symptoms would be expected to be acute but gradual over several minutes as the air gradually migrates from the abdomen in the thorax. However, in the case described here, both the subcutaneous emphysema and the pneumothoraces manifested hyperacutely, over seconds. In the preceding period the patient was very anxious with an exaggerated respiratory response and continuous retching during the procedure. We believe that the patient's response was the equivalent of intensive and continuous Valsalva manoeuvres. 

The Valsalva manoeuvre has been strongly associated with the hyperacute development of subcutaneous emphysema, pneumomediastinum, and more rarely pneumothorax [14–17]. We propose that the air escaping in the mediastinum and pleural space may use the same paths as described above, through deep fascial planes or diaphragmatic pores to migrate in the abdomen, explaining the presence of extraluminal air. Clinically, the patient would present with a combination of subcutaneous emphysema, pneumothorax, pneumomediastinum, pneumoretroperitoneum, and pneumoperitoneum.

## 4. Conclusion

Combining our observations with the preexisting theories, we propose that pneumothorax, pneumomediastinum, pneumoperitoneum, pneumoretroperitoneum, and subcutaneous emphysema during ERCP, in the absence of duodenal perforation, may be explained by leakage of air from a site of low resistance such as the sphincterotomy site, or as a result of copious Valsalva manoeuvres performed by a patient tolerating the procedure poorly.

## References

[1] Dickinson RJ, Davies S (1998). Post-ERCP pancreatitis and hyperamylasaemia: the role of operative and patient factors. *European Journal of Gastroenterology and Hepatology*.

[2] Loperfido S, Angelini G, Benedetti G (1998). Major early complications from diagnostic and therapeutic ERCP: a prospective multicenter study. *Gastrointestinal Endoscopy*.

[3] Savides T, Sherman S, Kadell B, Cryer H, Derezin M (1993). Bilateral pneumothoraces and subcutaneous emphysema after endoscopic sphincterotomy. *Gastrointestinal Endoscopy*.

[4] Markogiannakis H, Toutouzas KG, Pararas NV, Romanos A, Theodorou D, Bramis I (2007). Bilateral pneumothorax following endoscopic retrograde cholangiopancreatography: a case report. *Endoscopy*.

[5] Kocaman O, Sipahi M, Çubukçu A, Baykara ZN, Hülagü S (2009). Porous diaphragm syndrome after ERCP in a patient with bile duct stricture. *Turkish Journal of Gastroenterology*.

[6] Mosler P, Fogel EL (2007). Massive subcutaneous emphysema after attempted endoscopic retrograde cholangiopancreatography in a patient with a history of bariatric gastric bypass surgery. *Endoscopy*.

[7] Stapfer M, Selby RR, Stain SC (2000). Management of duodenal perforation after endoscopic retrograde cholangiopancreatography and sphincterotomy. *Annals of Surgery*.

[8] Mallery JS, Baron TH, Dominitz JA (2003). Complications of ERCP. *Gastrointestinal Endoscopy*.

[9] Silviera ML, Seamon MJ, Porshinsky B (2009). Complications related to endoscopic retrograde cholangiopancreatography: a comprehensive clinical review. *Journal of Gastrointestinal and Liver Diseases*.

[10] Howard TJ, Tan T, Lehman GA (1999). Classification and management of perforations complicating endoscopic sphincterotomy. *Surgery*.

[11] Enns R, Eloubeidi MA, Mergener K (2002). ERCP-related perforations: risk factors and management. *Endoscopy*.

[12] Masci E, Toti G, Mariani A (2001). Complications of diagnostic and therapeutic ERCP: a prospective multicenter study. *American Journal of Gastroenterology*.

[13] Aronson N, Flamm CR, Bohn RL, Mark DH, Speroff T (2002). Evidence-based assessment: patient, procedure, or operator factors associated with ERCP complications. *Gastrointestinal Endoscopy*.

[14] Panacek EA, Singer AJ, Sherman BW, Prescott A, Rutherford WF (1992). Spontaneous pneumomediastinum: clinical and natural history. *Annals of Emergency Medicine*.

[15] Pierce MJ, Weesner CL, Anderson AR, Albohm MJ (1998). Pneumomediastinum in a female track and field athlete: a case report. *Journal of Athletic Training*.

[16] Reddymasu S, Borhan-Manesh F, Jordan PA (2006). Spontaneous pneumomediastinum due to achalasia: a case report. *Southern Medical Journal*.

[17] Minocha A, Richards RJ (1991). Pneumomediastinum as a complication of upper gastrointestinal endoscopy. *Journal of Emergency Medicine*.

